# Parallel algorithm for convection–diffusion system based on least-squares procedure

**DOI:** 10.1186/s40064-016-3333-8

**Published:** 2016-10-01

**Authors:** Jiansong Zhang, Hui Guo, Hongfei Fu, Yanzhen Chang

**Affiliations:** 1 Department of Applied Mathematics, China University of Petroleum, Qingdao, 266580 China; 2 Department of Computational Mathematics, China University of Petroleum, Qingdao, 266580 China; 3 Department of Pure Mathematics, China University of Petroleum, Qingdao, 266580 China; 4 Department of Mathematics, Beijing University of Chemical Technology, Beijing, 100029 China

**Keywords:** Overlapping domain decomposition, Parallel subspace correction, Least-squares, Convection–diffusion system, 65M55, 65M60, 65M12, 65M15

## Abstract

Combining subspace correction method with least-squares finite element procedure, we construct a new overlapping domain decomposition parallel algorithm for solving the first-order time-dependent convection–diffusion system. This algorithm is fully parallel. We analyze the convergence of approximate solution, and study the dependence of the convergent rate on the spacial mesh size, time increment, iteration number and sub-domains overlapping degree. Both theoretical analysis and numerical results suggest that only one or two iterations are needed to reach to given accuracy at each time step.

## Background

In this paper, we consider the following initial-boundary value problem for time-dependent convection–diffusion system:

1 $$\left\{
\begin{array}{l} c \frac{\partial u}{\partial t}+\nabla \cdot \mathbf {\sigma }+qu = f, \quad x\in \varOmega ,\;\;0< t\le T,\\{\sigma }+A\nabla u+\mathbf {b}u = 0,\quad\,\, \,x\in \varOmega ,\;\;0<t\le T,\\ u=0,\qquad \qquad \qquad \,\, x\in \varGamma _D,\\ \mathbf{\sigma} \cdot \nu = 0, \qquad \qquad \,\,\,\, x\in \varGamma _N,\ \ 0\le t\le T,\\ u(x,0) = u_0(x),\qquad x\in \varOmega , \end{array}\right.$$

where $$\varOmega $$ is an open bounded domain $$\mathbf {R}^d$$$$(1\le d\le 3)$$, with a Lipschitz continuous boundary $$\varGamma =\varGamma _D\cup \varGamma _N$$; and $$\nu $$ is the unit vector normal to $$\varGamma _N$$; the flow field $$\mathbf {b}=(b_1,b_2,\ldots ,b_d)^T$$; the source term $$q=q(x,t)\ge 0$$ and exterior flow function $$f=f(x,t)$$ are some given functions; the coefficient $$c=c(x)$$ is positive function and the diffusion coefficient matrix $$A=(a(i,j))_{d\times d}$$ is a symmetric uniformly positive definite matrix, i.e., there exist some positive constants $$c_*$$ and $$a_*$$ such that

2 $$\begin{aligned} a_*\sum ^d_{i=1}\xi ^2_i\le \sum ^d_{i,j=1}a_{ij}(x)\xi _i\xi _j,\quad c_*\le c(x),\,\,\forall \xi \in \mathbf {R}^d,\,x\in \varOmega . \end{aligned}$$

This type of partial differential equation arises in many important fields, such as the mathematical modeling of aerodynamics, porous medium fluid flow, fluid dynamics (e.g. Euler equations, Navier-Stokes equations), meteorology, and semiconductor devices. Many numerical methods have been established to simulate this problem, for example, finite element and finite difference method, Eulerian–Lagrangian localized adjoint method Celia et al. (1990). The streamline diffusion finite element method Hughes and Brooks (1979), least-squares mixed element methods Yang (1999, 2000, 2002), Zhang and Guo (2012), Zhang et al. (2011) and Zhang (2009), and so on. Generally, these numerical procedures result in a large scale of algebraic system, so it is very important and useful to develop effective parallel algorithms both in engineering applications and mathematical analysis.

Recently domain decomposition parallel computation has become a powerful tool for solving a large scale system of partial differential equations. A lot of work has been done on domain decomposition parallel algorithms, for example, see Beilina (2016), Bramble et al. (1990, 1991), Cai (1989), Dolean et al. (2008, 2015), Dryja and Widlund (1987), Lu et al. (1991), Ma et al. (2009), Tarek (2008), Xu (1989, 1992, 2001) and Yang (2010). But many parallel algorithms based on overlapping domain decomposition are iterative algorithms so that many iteration steps are needed to reach given accuracy, which leads to much more global amount of computational work. On the basis of the idea of the parallel subspace correction method proposed by Xu (1989, 1992, 2001), the first author of this paper and Yang established a new parallel algorithm combined with characteristic finite element scheme, finite difference scheme and least-square scheme for one dimensional convection–diffusion problem in Zhang et al. (2011) and Zhang and Yang (2011a, b), where both theoretical analysis and numerical results suggest that when overlapping degree has a positive lower bound independent of mesh size, only one or two iterative times is needed to reach the optimal convergence precision at each time level.

In this paper, using the same technique as in Zhang et al. (2011), Zhang and Yang (2011) and Zhang and Yang (2011), we establish a new parallel algorithm for solving the convection–diffusion system. Here the arbitrary dimensional problem is considered, unlike in Zhang and Yang (2011) only one dimensional model was studied. And the different least-squares finite element scheme from the one in Zhang and Yang (2011) is used to obtain the optimal $L^2$-norm error estimate. The partition of unity is applied to distribute the corrections in the overlapping domains reasonably in this parallel algotithm. We analyze the convergence of approximate solution, and study the dependence of the convergent rate on the spacial mesh size, time increment, iteration number and sub-domains overlapping degree. Both theoretical analysis and numerical experiments indicate the full parallelization of the algorithms and very good approximate property.

## Parallel algorithm

Throughout this paper we use usual definitions and notations of Sobolev spaces as in Adams (1975). Let $W^{k,p}\,(\varOmega )\,(k\ge 0,\,1\le p\le \infty )$ be Sobolev spaces defined on $\varOmega$ with usual norms $\Vert \cdot \Vert _{W^{k,p}\,(\varOmega )}$ and $H^k(\varOmega )=W^{k,2}\,(\varOmega )$. Define inner products as follows:

$$\begin{aligned} (u,v)& =  \int _\varOmega u(x)v(x)dx\quad \forall \, u,v \in L^2(\varOmega ),\\ (\sigma ,\omega )&=  {\mathop {\sum }\limits ^d_{i=1}}(\sigma _i,\omega _i)\quad \forall \, \sigma , \omega \in [L^2(\varOmega )]^d,\quad 1\le d\le 3. \end{aligned}$$

Introduce the spaces $\mathcal {W}=\{\omega \in [L^2(\varOmega )]^d;\,\nabla \cdot {\omega }\in L^2(\varOmega ),\, \omega \cdot \nu =0\,\,\text {on} \,\,\varGamma _N\}$ and $\mathcal {V}=\{v\in H^1(\varOmega );\,\,v=0\,\,\text {on}\,\,\varGamma _D\}$. Make a time partition $0=t_0<t_1<\cdots<t_{M-1}<t_M=T$ and set $\tau _n=t_n-t_{n-1}$ and $\tau =\max \nolimits _{1\le n\le M}\tau _n$. Let $w^n(x)=w(x,t_n)$. By use of the difference technique with first-order accuracy to discretize the first-order system (1), we can rewrite the system (1) as follows [see Yang (1999)]

3 $$\begin{aligned}c(x)\bar{\partial }_tu^n(x)+\nabla \cdot \sigma ^n(x)+q^n(x)u^n(x)&=f^{n}(x)+R^n(x),\quad x\in \varOmega ,\nonumber \\ \bar{\partial }_t{\sigma }^n(x)+A(x)\nabla \bar{\partial }_tu^n(x)+\bar{\partial }_t(\mathbf {b}^n(x)u^n(x))&=0,\quad x\in \varOmega ,\nonumber \\ u^n(x)&=0,\quad x\in \varGamma _D,\\ \sigma ^n(x)\cdot \nu (x)&=0,\quad x\in \varGamma _N,\nonumber \\ u^0(x)&=u_0(x),\quad x\in \varOmega \end{aligned}$$

where

$$\begin{aligned} R^n(x)&= c(x)(\bar{\partial }_tu^n(x)-u_t(x))=O\left(\tau _n\frac{\partial ^2 u}{\partial t^2}\right),\\ \bar{\partial }_tu^n(x)& = (u^n-u^{n-1})/\tau _n. \end{aligned}$$

To construct parallel subspace correction algorithm, we firstly make a domain decomposition. Assume that $\{\varOmega '_i\}^N_{i=1}$ is a non-overlapping domain decomposition of $\varOmega$. In order to obtain an overlapping domain decomposition, we extend each subregion $\varOmega '_i$ to a larger region $\varOmega _i$ such that $\varOmega '_i\subset \varOmega _i\subset \varOmega$ and $dist(\partial \varOmega '_i \backslash \partial \varOmega ,\partial \varOmega _i \backslash \partial \varOmega )\ge H$ for each $1 \le i \le N$, where $H >0$ is called as overlapping degree. Let $\mathcal {T}_{h_u}$ and $\mathcal {T}_{h_\sigma }$ be two families of quasi-regular finite element partitions of the domain $\varOmega$ such that the elements in the partitions have the diameters bounded by $h_u$ and $h_\sigma$, respectively. Assume that $\mathcal {T}_{h_u,i}=\mathcal {T}_{h_u}\bigcap \varOmega _i$ and $\mathcal {T}_{h_\sigma ,i}=\mathcal {T}_{h_\sigma } \bigcap \varOmega _i$ just are one finite element partition of $\varOmega _i$ for $1 \le i \le N$. Let ${\mathcal W}_{h_\sigma }\subset \mathcal {W}$, and ${\mathcal V}_{h_u}\subset \mathcal {V}$ be piecewise *r*-degree and *k*-degree polynomial spaces defined on the partitions $\mathcal {T}_{h_\sigma }$ and $\mathcal {T}_{h_u}$, respectively.

Denote by $\tilde{A}$ the inverse of *A* and define a bilinear form

$$\begin{aligned} a_n((\sigma ,w),(\omega ,v))&=\left(\frac{1}{c}(cw+\tau _n(\nabla \cdot \mathbf {\sigma } +q^nw)),cv+\tau _n(\nabla \cdot \mathbf {\omega }+q^nv)\right)\\ &\quad +\tau _n(\tilde{A}(\sigma +A\nabla w+\mathbf b^nw),\omega +A\nabla v+\mathbf b^nv). \end{aligned}$$

Based on (3) and Yang (1999), we get the standard least-squares finite element procedure:

**Least-squares scheme***Given an initial approximation*$(\varrho ^0_h,w^0_h)\in {\mathcal W}_{h_\sigma }\times {\mathcal V}_{h_u}$. *For*$n=1,2,\ldots ,M$, *seek*$(\varrho ^n_h,w^n_h)\in {\mathcal W}_{h_\sigma }\times {\mathcal V}_{h_u}$*such that*

4 $$\begin{aligned}&a_n((\varrho ^n_h,w^n_h),(\omega _h,v_h))\nonumber \\ &\quad =\left( \frac{1}{c}(c w^{n-1}_h+\tau _nf^{n}),cv_h+\tau _n(\nabla \cdot \omega _h+q^nv_h)\right) \nonumber \\ &\quad \quad +\tau _n\left( \tilde{A}(\varrho ^{n-1}_h+A\nabla w^{n-1}_h+\mathbf b^nw^{n-1}_h),\omega _h+A\nabla v_h+\mathbf b^nv_h\right) ,\nonumber \\ &\quad \quad \quad \forall \,(\omega _h,v_h)\in {\mathcal W}_{h_\sigma }\times {\mathcal V}_{h_u}. \end{aligned}$$

In the following part of this section, we propose the parallel domain decomposition algorithm of the system (4). Define finite element sub-spaces:

$$\begin{aligned} {\mathcal V}^i_{h_u}=\left\{ v_h \in {\mathcal V}_{h_u}; \ \ v_h=0 \ \rm in \ \ \varOmega \backslash \varOmega _i \right\} , \ \ \ 1 \le i \le N \end{aligned}$$

and

$$\begin{aligned} {\mathcal W}^i_{h_\sigma }=\left\{ \sigma _h \in {\mathcal W}_{h_\sigma }; \ \ \sigma _h=0 \ \rm in \ \ \varOmega \backslash \varOmega _i \right\} , \ \ \ 1 \le i \le N. \end{aligned}$$

It is clear that

$$\begin{aligned} {\mathcal V}_{h_u}={\mathcal V}^1_{h_u}+{\mathcal V}^2_{h_u}+ \cdots + {\mathcal V}^N_{h_u} \end{aligned}$$

and

$$\begin{aligned} {\mathcal W}_{h_\sigma }={\mathcal W}^1_{h_\sigma }+{\mathcal W}^2_{h_\sigma }+ \cdots + {\mathcal W}^N_{h_\sigma }. \end{aligned}$$

It is easily seen that there exists a finite open covering family $\{O^i\}^N_{i=1}$ of the domain $\varOmega$ such that $O^i\cap \varOmega \subset \varOmega _i$. We know that there exists a partition of unity $\{\varphi _i\}^N_{i=1}$ (see Toselli and Widlund (2005), Lemma 3.4) such that

$$\begin{aligned} &(\text {a})\quad supp(\varphi _i)\subset O^i, \ \ 0\le \varphi _i\le 1,\ \ \ \Vert \varphi _i\Vert _{W^{r,\infty }}\le CH^{-r}, \ \ 1 \le i \le N;\\ & (\text {b})\quad \varphi _1+\varphi _2+ \cdots + \varphi _N=1 \ \rm in \ \ \varOmega . \end{aligned}$$

Let $\varphi ^i_{h_u}$ and $\varphi ^i_{h_\sigma }$ be the nodal piecewise linear interpolation of $\varphi _i$ on the finite element meshes ${\mathcal T}_{h_u}$ and ${\mathcal T}_{h_\sigma }$, and ${\mathcal I}_{h_u}$ and ${\mathcal I}_{h_\sigma }$ be the interpolating operators on $\mathcal {V}_{h_u}$ and $\mathcal {W}_{h_\sigma }$.

Based on (4), we formulate the parallel subspace correction algorithm.

**Parallel algorithm***Letmdenote the iteration number at each time step. Give an initial approximation*$(\sigma ^0_h,u^{0}_h)=(\varrho ^0_h,w^0_h) \in {\mathcal W}_{h_\sigma }\times {\mathcal V}_{h_u}$. For $n=1,\ 2,\ \ldots , M$, *seek*$(\sigma ^n_h,u^n_h)\in {\mathcal W}_{h_\sigma }\times {\mathcal V}_{h_u}$*by four steps:*

*Step 1. Set*$(\tilde{\sigma }^n_{0},\tilde{u}^n_{0})=(\sigma ^{n-1}_h,u^{n-1}_h)$ and $j:=1$.

*Step 2. For*$i=1,2,\ldots ,N,$*seek*$(\varepsilon ^i_{j},e^i_{j})\in {\mathcal W}^i_{h_\sigma }\times {\mathcal V}^i_{h_u}$, *in parallel, such that*

5 $$\begin{aligned}
&a_n\left( (\varepsilon ^i_{j},e^i_{j}),(\omega _h,v_h)\right) \nonumber
\\ &\quad =\left( \frac{1}{c}(c u^{n-1}_h+\tau _nf^{n}),c{\mathcal I}_{h_u}(\varphi _{h_u}^iv_h)\right. \nonumber
\\ & \qquad \left. +\tau _n( \nabla \cdot {\mathcal I}_{h_\sigma }(\varphi ^i_{h_\sigma }\omega _h) +q^n{\mathcal I}_{h_u}(\varphi ^i_{h_u}v_h))\vphantom{{\frac{1}{c}}}\right) \nonumber
\\ &\qquad +\tau _n\left( \tilde{A}(\sigma ^{n-1}_h+A\nabla u^{n-1}_h+\mathbf b^nu^{n-1}_h),{\mathcal I}_{h_\sigma }(\varphi ^i_{h_\sigma }\omega _h\right) \nonumber
\\ &\qquad \left. +\;A\nabla {\mathcal I}_{h_u}(\varphi ^i_{h_u}v_h)+\mathbf b^n{\mathcal I}_{h_u}(\varphi ^i_{h_u}v_h)\right) \nonumber
\\ &\qquad \ -a_n\left( (\widetilde{\sigma }^n_{j-1},\widetilde{u}^n_{j-1}),({\mathcal I}_{h_\sigma }(\varphi ^i_{h_\sigma }\omega _h),{\mathcal I}_{h_u}(\varphi ^i_{h_u}v_h))\right) ,\nonumber
\\ &\qquad\quad \forall \ (\omega _h,v_h) \in {\mathcal W}_{h_\sigma }\times {\mathcal V}_{h_u}. \end{aligned}$$

*Step 3. Set corrections*

6 $$\begin{aligned} \tilde{\sigma }^n_j=\tilde{\sigma }^n_{j-1}+\sum \limits ^N_{i=1}\varepsilon ^i_{j}, \quad \tilde{u}^n_j=\tilde{u}^n_{j-1}+\sum \limits ^N_{i=1}e^i_{j}. \end{aligned}$$

*Step 4. If*$j < m$, *then set*$j:=j+1$*and return the step 2; or set*

$$\begin{aligned} \ \sigma ^n_h=\tilde{\sigma }^n_m, \quad u^n_h=\tilde{u}^n_m \end{aligned}$$

*and then return back to the first step to start iteration at the next time step.*

## Some lemmas and main result

In the following sections, we denote by *K* and $$\delta $$ some general constants and small positive constants independent of the mesh parameters *H*, $$h_\sigma $$$$h_u$$ and $$\tau $$, which may be different at different occurrences. Let

$$\begin{aligned} \Vert (\omega ,v)\Vert ^2_{a_n}&=\left(\frac{1}{c}(cv+\tau _n(\nabla \cdot {\omega } +q^nv)),cv+\tau _n(\nabla \cdot \mathbf {\omega }+q^nv)\right)\\ &\quad +\tau _n\left(\tilde{A}(\omega +A\nabla v+\mathbf b^nv),\omega +A\nabla v+\mathbf b^nv\right). \end{aligned}$$

In order to analyze the convergence of parallel algorithm, we introduce projection operators $P^i_{h_\sigma }{:}\,{\mathcal W}_{h_\sigma }\rightarrow {\mathcal W}^i_{h_\sigma }$ and $Q^i_{h_u}:\,{\mathcal V}_{h_u}\rightarrow {\mathcal V}^i_{h_u}$ such that

$$\begin{aligned}&a_n\left((P^i_{h_\sigma }\omega ,Q^i_{h_u}v),(\omega _h,v_h)\right)=a_n\left((\omega ,v),(\omega _h,v_h\right)),\\ &\quad \forall \ \ (\omega _h,v_h)\in {\mathcal W}^i_{h_\sigma }\times {\mathcal V}^i_{h_u}\,\,i=1,2\ldots ,N. \end{aligned}$$

Now, we give some important lemmas which are used to analyze the convergence of parallel algorithm.

We assume that finite element spaces $\mathcal {W}_{h_\sigma }$ and $\mathcal {V}_{h_u}$ have the inverse property and approximate properties [see Ciarlet (1978)] that there exist some integers $r,\,r_1,\,k>0$, such that, for $1\le q\le \infty$ and $\forall \,\omega \in H(\text {div};\varOmega )\cap [W^{r+1,q}(\varOmega )]^d$,

$$\begin{aligned}&\inf _{\omega _h\in \mathcal {W}_{h_\sigma }}\Vert \omega -\omega _h\Vert _{[L^q(\varOmega )]^d}\le Kh^{r+1}_\sigma \Vert \omega \Vert _{[W^{r+1,q}(\varOmega )]^d},\\ &\inf _{\omega _h\in \mathcal {W}_{h_\sigma }}\Vert \nabla \cdot (\omega -\omega _h)\Vert _{L^q(\varOmega )}\le Kh^{r_1}_\sigma \Vert \nabla \cdot \omega \Vert _{W^{r_1,q}(\varOmega )},\\ &\inf _{v_h\in \mathcal {V}_{h_u}}\Vert v-v_h\Vert _{L^q(\varOmega )}\le Kh^{k+1}_u\Vert v\Vert _{W^{k+1,q}(\varOmega )},\forall \, v\in L^2(\varOmega )\cap W^{k+1,q}(\varOmega ). \end{aligned}$$

Based on Theorem 3.3 in Yang (1999), the following result can be read:

### **Lemma 1**

*Let*$(\sigma ,u)$*and*$(\varrho ^n_h,w^n_h)$*be the solutions of* (1) *and least-squares scheme, respectively. Then there holds the a priori error estimate*

7 $$\begin{aligned} \max _n\Vert u^n-w^n_h\Vert _{L^2(\varOmega )} +\max _n\Vert \sigma ^n-\varrho ^n_h\Vert _{[L^2(\varOmega )]^d}\le K\{h^{k+1}_u+h^{r_1}_\sigma +\tau \}. \end{aligned}$$

### **Lemma 2**

[See Yang (2001)] *For any function*$\varphi \in W^{1,\infty }(\varOmega )$*and*$\omega _h\in \mathcal {W}_{h_\sigma }$, *we have the following estimate*

$$\begin{aligned} \Vert \varphi \omega _h-\mathcal {I}_{h_\sigma } (\varphi \omega _h)\Vert _{[L^2(\varOmega )]^d} \le Kh_\sigma \min (\Vert \varphi \Vert _{W^{1,\infty }(\varOmega )}\Vert \omega _h\Vert _{[L^2(\varOmega )]^d}, \Vert \varphi \Vert _{H^1(\varOmega )}\Vert \omega _h\Vert _{[L^\infty (\varOmega )]^d}), \end{aligned}$$

*where*$$d=1,2,3.$$

### **Lemma 3**

*For*$1\le i\le N$, *we have*

8 $$\begin{aligned}\Vert (\mathcal {I}-\mathcal {I}_{h_\sigma })(\varphi ^i_{h_\sigma }{\omega _h})\Vert _{[L^2(\varOmega )]^d}&\le K\frac{h_\sigma }{H}\Vert {\omega _h}\Vert _{[L^2(\varOmega )]^d},\quad \forall \,\omega _h\in \mathcal {W}_{h_\sigma },\nonumber \\ \Vert (\mathcal {I}-\mathcal {I}_{h_u})(\varphi ^i_{h_u}v_h)\Vert _{L^2(\varOmega )}&\le K\frac{h_u}{H}\Vert v_h\Vert _{L^2(\varOmega )}, \quad \forall \, v_h\in \mathcal {V}_{h_u}. \end{aligned}$$

### *Proof*

Using Lemma 2, we know that

$$\begin{aligned} \Vert (\mathcal {I}-\mathcal {I}_{h_\sigma })(\varphi ^i_{h_\sigma }\omega _h)\Vert _{[L^2(\varOmega )]^d} \le Kh_\sigma \Vert \varphi ^i_{h_\sigma }\Vert _{W^{1,\infty }(\varOmega )}\Vert {\omega _h}\Vert _{[L^2(\varOmega )]^d}\le K\frac{h_\sigma }{H}\Vert {\omega _h}\Vert _{[L^2(\varOmega )]^d}. \end{aligned}$$

This is the first inequality of (8).

In addition, by using the technique of Theorem 3.1 in Yang (2001), we can easily obtain

$$\begin{aligned}&\Vert (\mathcal {I}-\mathcal {I}_{h_u})(\varphi ^i_{h_u}v_h)\Vert _{L^2(\varOmega )}\\ &\qquad \le Kh_u\min (\Vert \varphi ^i_{h_u}\Vert _{W^{1,\infty }(\varOmega )}\Vert v_h\Vert _{L^2(\varOmega )}, \Vert \varphi ^i_{h_u}\Vert _{H^{1}(\varOmega )}\Vert v_h\Vert _{L^\infty (\varOmega )})\\ &\qquad \le Kh_u\Vert \varphi ^i_{h_u}\Vert _{W^{1,\infty }(\varOmega )}\Vert v_h\Vert _{L^2(\varOmega )}\le K\frac{h_u}{H}\Vert v_h\Vert _{L^2(\varOmega )}. \end{aligned}$$

That is the second inequality of (8). The proof of Lemma 3 is completed. $\square$

### **Lemma 4**

*The following estimate*

9 $$\begin{aligned}&\left| \ a_n\left( (\psi ,w),(\omega ,v))-\sum \limits ^N_{i=1}a_n(( \psi ,w),({\mathcal I}_{h_\sigma }(\varphi _{h_\sigma }^iP^i_{h_\sigma }\omega ),{\mathcal I}_{h_u}(\varphi _{h_u}^iQ^i_{h_u}v)\right) \right| \nonumber \\ &\quad \le K\left(\frac{h}{H}+\frac{\sqrt{\tau }}{H}\right)\Vert ( \psi ,w)\Vert _{a_n}\Vert (\omega _h,v_h)\Vert _{a_n} \end{aligned}$$

*holds for each*$( \psi ,w)$*and*$( \omega ,v)$*in*${\mathcal W}_{h_\sigma } \times {\mathcal V}_{h_u}$.

### *Proof*

It is easily seen that

$$\begin{aligned}&a_n\left((\psi ,w),({\mathcal I}_{h_\sigma }(\varphi ^i_{h_\sigma }P^i_{h_\sigma }\omega ), {\mathcal I}_{h_u}(\varphi ^i_{h_u} Q^i_{h_u}v))\right)\\ &\quad =a_n\left( (\psi ,w),(\varphi ^i_{h_\sigma }P^i_{h_\sigma }\omega , \ \varphi ^i_{h_u} Q^i_{h_u}v)\right) \\ &\qquad +a_n\left((\psi ,w),(({\mathcal I}_{h_\sigma }-{\mathcal I})(\varphi ^i_{h_\sigma }P^i_{h_\sigma }\omega ), ({\mathcal I}_{h_u}-{\mathcal I})(\varphi ^i_{h_u} Q^i_{h_u}v))\right), \end{aligned}$$

and

$$\begin{aligned}&a_n\left( (\psi ,w),(\varphi ^i_{h_\sigma }P^i_{h_\sigma }\omega ,\ \varphi ^i_{h_u} Q^i_{h_u}v)\right) \\ &\quad =a_n\left( (P^i_{h_\sigma }(\varphi ^i_{h_\sigma }\psi ),Q^i_{h_u}(\varphi ^i_{h_u}w)),(\omega ,\ v)\right) \\ &\qquad +\tau _n \left[ \left(\frac{1}{c}(cw+\tau _n(\nabla \cdot \psi +q^nw)), (P^i_{h_\sigma }\omega )\nabla \varphi _{h_\sigma }^i\right)\right. \\ &\qquad -\left(\frac{1}{c}\psi \nabla \varphi _{h_\sigma }^i,cQ^i_{h_u}v+\tau _n(\nabla \cdot (P^i_{h_\sigma }\omega \right)+q^nQ^i_{h_u}v))\\ &\qquad +(\tilde{A}(\psi +A\nabla w+\mathbf {b}^nw), A \nabla \varphi ^h_iQ^i_{h_u}v)\\ &\qquad \left. -(\nabla \varphi _{h_u}^iw,P^i_{h_\sigma }\omega +A\nabla (Q^i_{h_u}v)+\mathbf {b}^nQ^i_{h_u}v)\vphantom{\frac{1}{c}}\right] \end{aligned}$$

and

$$\begin{aligned} a_n\left( (\psi ,w),(\omega ,\ v)\right) =\sum \limits ^N_{i=1}a_n\left( (\varphi ^i_{h_\sigma }\psi ,\varphi ^i_{h_u}w),(\omega ,\ v)\right) . \end{aligned}$$

Hence we have

10 $$\begin{aligned}&a_n\left( (\psi ,w),(\omega ,\ v)\right) -\sum \limits ^N_{i=1}a_n((\psi ,w),({\mathcal I}_{h_\sigma }(\varphi ^i_{h_\sigma }P^i_{h_\sigma }\omega ), {\mathcal I}_{h_u}(\varphi ^i_{h_u} Q^i_{h_u}v)))\nonumber \\ &\quad =\sum \limits ^N_{i=1}a_n\left( (({\mathcal I}-P^i_{h_\sigma })(\varphi ^i_{h_\sigma }\psi ), ({\mathcal I}-Q^i_{h_u})(\varphi ^i_{h_u}w)),(\omega ,\ v)\right) \nonumber \\ &\qquad -\sum \limits ^N_{i=1}a_n((\psi ,w),(({\mathcal I}_{h_\sigma }-{\mathcal I})(\varphi ^i_{h_\sigma }P^i_{h_\sigma }\omega ), ({\mathcal I}_{h_u}-{\mathcal I})(\varphi ^i_{h_u} Q^i_{h_u}v)))\nonumber \\ &\qquad\ -\tau _n \sum \limits ^N_{i=1}\left[ \left(\frac{1}{c}(cw+\tau _n(\nabla \cdot \psi +q^nw)), (P^i_{h_\sigma }\omega )\nabla \varphi _{h_\sigma }^i \right)\right. \nonumber \\ &\qquad \ -\left(\frac{1}{c}\psi \nabla \varphi _{h_\sigma }^i,cQ^i_{h_u}v+\tau _n(\nabla \cdot (P^i_{h_\sigma }\omega )+q^nQ^i_{h_u}v) \right)\nonumber \\ &\qquad \ +(\tilde{A}(\psi +A\nabla w+\mathbf {b}^nw), A \nabla \varphi ^i_{h_u}Q^i_{h_u}v)\nonumber \\ &\qquad \left. -\;(\nabla \varphi _{h_u}^iw,P^i_{h_\sigma }\omega +A\nabla (Q^i_{h_u}v)+\mathbf {b}^nQ^i_{h_u}v)\right] . \end{aligned}$$

Noting that

$$\begin{aligned}
&\Vert ({\mathcal I}_{h_\sigma }-{\mathcal I})(\varphi ^i_{h_\sigma
}P^i_{h_\sigma }\omega ), ({\mathcal I}_{h_u}-{\mathcal I})(\varphi
^i_{h_u} Q^i_{h_u}v)\Vert _{a_n}
\\ &\quad \le K \{\Vert
c({\mathcal I}_{h_u}-{\mathcal I})(\varphi ^i_{h_u} Q^i_{h_u}v)\Vert
_{L^2(\varOmega _i)} +\tau _n\Vert \nabla \cdot ({\mathcal
I}_{h_\sigma }-{\mathcal I})(\varphi ^i_{h_\sigma }P^i_{h_\sigma
}\omega )\Vert _{L^2(\varOmega _i)}
\\ &\qquad\ +\tau _n\Vert
q^n({\mathcal I}_{h_u}-{\mathcal I})(\varphi ^i_{h_u}
Q^i_{h_u}v)\Vert _{L^2(\varOmega _i)} +\sqrt{\tau _n}\ \left[ \
\Vert ({\mathcal I}_{h_\sigma }-{\mathcal I})(\varphi ^i_{h_\sigma
}P^i_{h_\sigma }\omega )\Vert _{L^2(\varOmega _i)}\right.
\\ &\qquad \left. \left.+\;\Vert A\nabla (({\mathcal I}_{h_u}-{\mathcal I})(\varphi
^i_{h_u} Q^i_{h_u}v)\Vert _{L^2(\varOmega _i)} +\Vert \mathbf
{b}^n({\mathcal I}_{h_u}-{\mathcal I})(\varphi ^i_{h_u}
Q^i_{h_u}v)\Vert _{L^2(\varOmega _i)} \right] \right\}
\\ &\quad \le
K\left\{(1+\tau _n)\frac{h_u}{H}\Vert Q^i_{h_u}v\Vert
_{L^2(\varOmega _i)} \tau _n\frac{1}{h_\sigma }\frac{h_\sigma
}{H}\Vert P^i_{h_\sigma }\omega \Vert _{[L^2(\varOmega _i)]^d}\right.
\\
&\qquad \left.+\sqrt{\tau _n} \left[ \frac{h_\sigma }{H}\Vert
P^i_{h_\sigma }\omega \Vert _{[L^2(\varOmega _i)]^d}
+\frac{1}{h_u}\frac{h_u}{H}\Vert Q^i_{h_u}v\Vert _{L^2(\varOmega
_i)}+\frac{h_u}{H}\Vert Q^i_{h_u}v\Vert _{L^2(\varOmega _i)}\right]
\right\}
\\ &\quad\le K\left(\frac{h}{H}+\frac{\sqrt{\tau
}}{H}\right) \left\{\ \Vert Q^i_{h_u}v\Vert _{L^2(\varOmega
_i)}+\sqrt{\tau _n}\ \left[ \Vert P^i_{h_\sigma }\omega \Vert
_{[L^2(\varOmega _i)]^d}+\Vert Q^i_{h_u}v\Vert _{L^2(\varOmega _i)}\
\right] \right\}\\ & \quad \le K\left(\frac{h}{H}+\frac{\sqrt{\tau
}}{H}\right) \Vert (P^i_{h_\sigma }\omega ,Q^i_{h_u}v)\Vert
_{a_n,\varOmega _i}, \end{aligned}
$$

we have

$$\begin{aligned}&\left|\sum\limits ^N_{i=1}a_n((\psi ,w),(({\mathcal I}_{h_\sigma }-{\mathcal I})(\varphi ^i_{h_\sigma }P^i_{h_\sigma }\omega ), ({\mathcal I}_{h_u}-{\mathcal I})(\varphi ^i_{h_u} Q^i_{h_u}v))) \right| \\ &\quad \le K\left(\frac{h}{H}+\frac{\sqrt{\tau }}{H}\right) \Vert (\psi ,w)\Vert _{a_n}\left[ \sum \limits ^N_{i=1} \Vert (P^i_{h_\sigma }\omega ,Q^i_{h_u}v)\Vert ^2_{a_n,\varOmega _i}\right] ^{1/2}, \\ &\left|\sum \limits ^N_{i=1}a_n\left( (({\mathcal I}-P^i_{h_\sigma })(\varphi ^i_{h_\sigma }\psi ), ({\mathcal I}-Q^i_{h_u})(\varphi ^i_{h_u}w)),(\omega ,\ v)\right)  \right|  \\ &\quad =\left| \sum \limits ^N_{i=1}a_n\left( (({\mathcal I}-{\mathcal I}_{h_\sigma })(\varphi ^i_{h_\sigma }\psi ), ({\mathcal I}-{\mathcal I}_{h_u})(\varphi ^i_{h_u}w)),(({\mathcal I}-P^i_{h_\sigma })\omega ,\ ({\mathcal I}-Q^i_{h_u})v)\right)\right|  \\ &\quad \le K\left(\frac{h}{H}+\frac{\sqrt{\tau }}{H}\right) \Vert (\psi ,w)\Vert _{a_n}\left[ \sum \limits ^N_{i=1} \Vert (({\mathcal I}-P^i_{h_\sigma })\omega,({\mathcal I}-Q^i_{h_u})v)\Vert ^2_{a_n,\varOmega _i}\right] ^{1/2} \end{aligned} $$

and

$$\begin{aligned}&\tau _n\sum \limits ^N_{i=1}\left[\left| \left(\frac{1}{c}(cw+\tau _n(\nabla \cdot \psi +q^nw)), (P^i_{h_\sigma }\omega )\nabla \varphi _{h_\sigma }^i\right)\right|\right. \\ &\qquad +\left| \left(\frac{1}{c}\psi \nabla \varphi _{h_\sigma }^i,cQ^i_{h_u}v+\tau _n(\nabla \cdot (P^i_{h_\sigma }\omega )+q^nQ^i_{h_u}v)\right)\right| \\ &\qquad +\left| (\tilde{A}(\psi +A\nabla w+\mathbf {b}^nw), A \nabla \varphi ^i_{h_u}Q^i_{h_u}v)\right| \\ &\qquad +\left. \left| (\nabla \varphi _{h_u}^iw,P^i_{h_\sigma }\omega +A\nabla (Q^i_{h_u}v)+\mathbf {b}^nQ^i_{h_u}v)\right| \vphantom{\sum \limits ^N_{i=1}}\right] \\ &\quad \le K\frac{\sqrt{\tau }_n}{H}\left[ \ \sum \limits ^N_{i=1} \Vert (\psi ,w)\Vert _{a_n}\Vert (P^i_{h_\sigma }\omega ,Q^i_{h_u}v)\Vert ^2_{a_n,\varOmega _i}\ \right] ^{1/2}. \end{aligned}$$

Substituting these estimates into (10) leads to (9). This ends the proof of Lemma 4. $\square$

For parallel algorithm , we have the following convergence result:

### **Theorem 1**

*Let*$(\sigma ,u)$*and*$(\sigma ^n_h,u^n_h)$*are the solutions of the system* (1) *and parallel algorithm, respectively. If*$h^{2m}=O(\tau )$, *then there holds the following a priori error* estimate

11 $$\begin{aligned}&{\max _n\Vert u^n-u^n_h\Vert _{L^2(\varOmega )}+\max _n\Vert \sigma ^n-\sigma _h^n\Vert _{[L^2(\varOmega )]^d}} \nonumber \\ &\qquad \le K\left\{\left(\frac{h^2}{H^2}+\frac{\tau }{H^2}\right)^\frac{m}{2}+h^{k+1}_u+h^{r_1}_\sigma +\tau \right\}, \end{aligned}$$

*where*$$h=\max (h_\sigma ,h_u)$$.

## Proof of Theorem 1

It is easily seen that parallel algorithm is also equivalent to use an iteration with initial values $({\sigma }^{n-1}_h,u^{n-1}_h)$ to solve the following equation: $(\hat{\sigma }^{n}_h,\hat{u}^n_h) \in {\mathcal W}_{h_\sigma } \times {\mathcal V}_{h_u}$ such that for any $(\omega _h,v_h) \in {\mathcal W}_{h_\sigma } \times {\mathcal V}_{h_u}$

12 $$\begin{aligned}&a_n((\hat{\sigma }^{n}_h,\hat{u}^n_h),(\omega _h,v_h)) =\left( \frac{1}{c}(c u^{n-1}_h+\tau _nf^{n}),cv_h+\tau _n(\nabla \cdot {\omega }_h+q^nv_h)\right) \nonumber +\tau _n\left( \tilde{A}(\sigma ^{n-1}_h+A\nabla u^{n-1}_h+\mathbf b^{n-1}u^{n-1}_h),\omega _h+A\nabla v_h+\mathbf b^nv_h\right) . \end{aligned}$$

From (12) we have

13 $$\begin{aligned}a_n(({\sigma }^{n}_h,u^n_h),(\omega _h,v_h)) &=\left( \frac{1}{c}(c u^{n-1}_h+\tau _nf^{n}),cv_h+\tau _n(\nabla \cdot {\omega }_h+q^nv_h)\right) \nonumber \\ & \quad +\tau _n\left( \tilde{A}(\sigma ^{n-1}_h+A\nabla u^{n-1}_h+\mathbf b^{n-1}u^{n-1}_h),\omega _h+A\nabla v_h+\mathbf b^nv_h\right) \nonumber \\ & \quad +a_n(({\sigma }^{n}_h-\hat{\sigma }^{n}_h,u^n_h-\hat{u}^n_h),(\omega _h,v_h)). \end{aligned}$$

Let $$\theta ^n=u^n_h-w^n_h,$$$$\rho ^n=w^n_h-u^n,$$$$\pi ^n=\sigma ^n_h-\varrho ^n_h$$ and $$\eta ^n=\varrho ^n_h-\sigma ^n.$$ Subtracting (4) from (13), we can get

14 $$\begin{aligned}a_n((\pi ^n,\theta ^n),(\omega _h,v_h)) &=\left( \theta ^{n-1},cv_h+\tau _n(\nabla \cdot {\omega }_h+q^nv_h)\right) \nonumber \\ & \quad +\tau _n\left( \tilde{A}(\pi ^{n-1}+A\nabla \theta ^{n-1}+\mathbf b^{n-1}\theta ^{n-1}),\omega _h+A\nabla v_h+\mathbf b^nv_h\right) \nonumber \\ & \quad +a_n(({\sigma }^{n}_h-\hat{\sigma }^{n}_h,u^n_h-\hat{u}^n_h),(\omega _h,v_h)). \end{aligned}$$

### **Lemma 5**

*For parallel algorithm, we have the estimate*

15 $$\begin{aligned} \Vert ({\sigma }^n_h-\hat{\sigma }^{n}_h,u^n_h-\hat{u}^n_h)\Vert _{a_n}\le K\left(\frac{h^2}{H^2}+\frac{\tau }{H^2}\right)^{\frac{m}{2}}\Vert ({\sigma }^{n-1}_h-\hat{\sigma }^{n}_h, u^{n-1}_h-\hat{u}^n_h)\Vert _{a_n}. \end{aligned}$$

### *Proof*

From (5), we have

16 $$\begin{aligned}a_n((\varepsilon ^i_{j},e^i_{j}),(\omega _h,v_h)) &=a_n((\varepsilon ^i_{j},e^i_{j}),(P^i_{h_\sigma }\omega _h,Q^i_{h_u}v_h)) \nonumber \\ &=a_n((\hat{\sigma }^{n}-\tilde{\sigma }^{n}_{j-1}, \hat{u}^n-\tilde{u}^{n}_{j-1}),({\mathcal I}_{h_\sigma }(\varphi ^i_{h_\sigma }P^i_{h_\sigma }\omega _h), {\mathcal I}_{h_u}(\varphi ^i_{h_u}Q^i_{h_u}v_h))). \end{aligned}$$

In addition, from parallel algorithm we can obtain the following equation

17 $$\begin{aligned}&a_n((\tilde{\sigma }^n_j-\hat{\sigma }^{n}_h,\tilde{u}^n_j-\hat{u}^n_h),(\omega _h,v_h)) \nonumber \\ &\quad =a_n((\tilde{\sigma }^n_{j-1}-\hat{\sigma }^{n}_h,\tilde{u}^n_{j-1}-\hat{u}^n_h),(\omega _h,v_h))+a_n\left(\left(\sum \limits ^N_{i=1}\varepsilon ^i_{j},\sum \limits ^N_{i=1}e^i_{j}\right),(\omega _h,v_h)\right) \nonumber \\ &\quad =a_n((\tilde{\sigma }^n_{j-1}-\hat{\sigma }^{n}_h,\tilde{u}^n_{j-1}-\hat{u}^n_h),(\omega _h,v_h)) \nonumber \\ &\qquad +a_n((\hat{\sigma }^{n}_h-\tilde{\sigma }^{n}_{j-1}, \hat{u}^n_h-\tilde{u}^{n}_{j-1}),\left(\sum \limits ^N_{i=1}{\mathcal I}_{h_\sigma }(\varphi ^i_{h_\sigma }P^i_{h_\sigma }\omega _h), \sum \limits ^N_{i=1}{\mathcal I}_{h_u}(\varphi ^i_{h_u}Q^i_{h_u}v_h))\right). \end{aligned}$$

Taking $(\omega _h,v_h)=(\tilde{\sigma }^n_j-\hat{\sigma }^{n}_h,\tilde{u}^n_j-\hat{u}^n_h)$ in (17) and using Lemma 4, we have

18 $$\begin{aligned} \Vert (\tilde{\sigma }^n_{j}-\hat{\sigma }^{n}_h,\tilde{u}^n_j-\hat{u}^n_h)\Vert ^2_{a_n}\le K\left(\frac{h^2}{H^2}+\frac{{\tau }}{H^2}\right)\Vert (\tilde{\sigma }^{n}_{j-1}-\hat{\sigma }^{n}_h,\tilde{u}^{n}_{j-1}-\hat{u}^n_h)\Vert ^2_{a_n}. \end{aligned}$$

Thus, we have

19 $$\begin{aligned} \Vert (\tilde{\sigma }^n_{m}-\hat{\sigma }^{n}_h,\tilde{u}^n_m-\hat{u}^n_h)\Vert ^2_{a_n}\le K\left(\frac{h^2}{H^2}+\frac{{\tau }}{H^2}\right)^m\Vert (\tilde{\sigma }^{n}_{0}-\hat{\sigma }^{n}_h,\tilde{u}^{n}_{0}-\hat{u}^n_h)\Vert ^2_{a_n}. \end{aligned}$$

That is the inequality (15). This ends the proof of Lemma 5. $\square$

Hence, we need to estimate the bounds of ${\sigma }^{n-1}_h-\hat{\sigma }^{n}_h$ and $u^{n-1}_h-\hat{u}^n_h$.

### **Lemma 6**

*For parallel algorithm, we have the following estimate*

20 $$\begin{aligned}&\Vert (\hat{\sigma }^{n}_h-{\sigma }^{n-1}_h,\hat{u}^n_h-u^{n-1}_h)\Vert ^2_{a_n} \nonumber \\ &\quad \le K\tau _n\left\{ \int ^{t^n}_{t^{n-1}}\left\Vert \left(\frac{\partial \varrho _h}{\partial t},\frac{\partial w_h}{\partial t}\right)\right\Vert ^2_{a_n}\text {d}t+\tau _n[\Vert \nabla \cdot \pi ^{n-1}\Vert ^2_{L^2(\varOmega )}+\Vert \theta ^{n-1}\Vert ^2_{L^2(\varOmega )}]\right\} . \end{aligned}$$

### *Proof*

From (14) we have

21 $$\begin{aligned}&a_n((\hat{\sigma }^{n}_h-{\sigma }^{n-1}_h,\hat{u}^n_h-u^{n-1}_h),(\omega _h,v_h)) \nonumber \\ &\quad =a_n(({\varrho }^{n}_h-\varrho ^{n-1}_h,w^n_h-w^{n-1}_h),(\omega _h,v_h)) \nonumber \\ &\quad \quad -\tau _n\left( \frac{1}{c}(\nabla \cdot \pi ^{n-1}+q^n\theta ^{n-1}),cv_h+\tau _n(\nabla \cdot {\omega }_h+q^nv_h)\right) \nonumber \\ &\quad \quad -\tau _n\left( \tilde{A}(\mathbf b^{n}-\mathbf b^{n-1})\theta ^{n-1}),\omega _h+A\nabla v_h+\mathbf b^nv_h\right) \end{aligned}$$

Taking $(\omega _h,v_h)=(\hat{\sigma }^{n}_h-{\sigma }^{n-1}_h,\hat{u}^n_h-u^{n-1}_h)$ in (21) and using the inequality $ab\le \frac{1}{\delta }a^2+\delta b^2$, we can obtain

$$\begin{aligned}&\Vert (\hat{\sigma }^{n}_h-{\sigma }^{n-1}_h,\hat{u}^n_h-u^{n-1}_h)\Vert ^2_{a_n}\\ &\quad \le K\tau _n\left\{ \int ^{t^n}_{t^{n-1}}\left\Vert \left(\frac{\partial \varrho _h}{\partial t},\frac{\partial w_h}{\partial t}\right)\right\Vert ^2_{a_n}\text {d}t +\tau _n[\Vert \nabla \cdot \pi ^{n-1}\Vert ^2_{L^2(\varOmega )}+\Vert \theta ^{n-1}\Vert ^2_{L^2(\varOmega )}]\right\} \\ &\quad \quad +\delta \Vert (\hat{\sigma }^{n}_h-{\sigma }^{n-1}_h,\hat{u}^n_h-u^{n-1}_h)\Vert ^2_{a_n} \end{aligned}$$

Hence, when we choose sufficiently small $\delta$, we can obtain the estimate (20). This ends the proof of Lemma 6. $\square$

Finally, we prove Theorem 1.

### *Proof*

Let $(\omega _h,v_h)=(\pi ^n,\theta ^n-\theta ^{n-1})$ in (14), we have

$$\begin{aligned}&a_n((\pi ^n,\theta ^n-\theta ^{n-1}),(\pi ^n,\theta ^n-\theta ^{n-1}))\\ &\quad =\tau _n\left( \tilde{A}(\pi ^{n-1}-\pi ^n),\pi ^n+A\nabla (\theta ^n-\theta ^{n-1})+\mathbf {b}^{n}(\theta ^n-\theta ^{n-1})\right) \\ &\quad \quad -\tau _n\left( \frac{1}{c}(\nabla \cdot \pi ^n+q^n\theta ^{n-1}),c(\theta ^n-\theta ^{n-1})+\tau _n(\nabla \cdot \pi ^n+q^n(\theta ^n-\theta ^{n-1}))\right) \\ &\quad \quad +\tau _n\left( \tilde{A}(\mathbf {b}^{n-1}-\mathbf {b}^n)\theta ^{n-1},\pi ^n+A\nabla (\theta ^n-\theta ^{n-1})+\mathbf {b}^n(\theta ^n-\theta ^{n-1})\right) \\ &\quad \quad +a_n(({\sigma }^{n}_h-\hat{\sigma }^{n}_h,u^n_h-\hat{u}^n_h),(\pi ^n,\theta ^n-\theta ^{n-1})). \end{aligned}$$

Since

$$\begin{aligned}&a_n((\pi ^n,\theta ^n-\theta ^{n-1}),(\pi ^n,\theta ^n-\theta ^{n-1}))-\tau _n(\tilde{A}(\pi ^{n-1}-\pi ^n),\pi ^n)\\ &\quad =(c(\theta ^n-\theta ^{n-1}),\theta ^n-\theta ^{n-1})+\tau _n\left[ (A\nabla (\theta ^n-\theta ^{n-1}),\nabla (\theta ^n-\theta ^{n-1}))\right. \\ &\quad \quad +(\tilde{A}\mathbf b^n(\theta ^n-\theta ^{n-1}),\theta ^n-\theta ^{n-1}) +\tau _n\left(\frac{1}{c}\nabla \cdot \pi ^n,\nabla \cdot \pi ^n\right)\\ &\left. \quad \quad +\;\tau _n\left(\frac{1}{c}q^n(\theta ^n-\theta ^{n-1}),q^n(\theta ^n-\theta ^{n-1})\right)\right] +\tau _n(\tilde{A}\pi ^n,\pi ^n)\\ &\quad \quad +\frac{\tau _n}{2}[(\tilde{A}\pi ^n,\pi ^n)-(\tilde{A}\pi ^{n-1},\pi ^{n-1})+(\tilde{A}(\pi ^n-\pi ^{n-1}),\pi ^n-\pi ^{n-1})]\\ &\quad \quad +2\tau _n\left( \frac{1}{c}(c(\theta ^n-\theta ^{n-1})+\tau _n\nabla \cdot \pi ^n),q^n(\theta ^n-\theta ^{n-1})\right) \\ &\quad \quad +2\tau _n(\tilde{A}+\nabla (\theta ^n-\theta ^{n-1}),\mathbf b^n(\theta ^n-\theta ^{n-1})), \end{aligned}$$

we have

22 $$\begin{aligned}&(c(\theta ^n-\theta ^{n-1}),\theta ^n-\theta ^{n-1})+\tau _n\left[ (A\nabla (\theta ^n-\theta ^{n-1}),\nabla (\theta ^n-\theta ^{n-1}))\right. \nonumber \\ &\quad \quad +(\tilde{A}\mathbf b^n(\theta ^n-\theta ^{n-1}),\theta ^n-\theta ^{n-1}) +\tau _n\left(\frac{1}{c}\nabla \cdot \pi ^n,\nabla \cdot \pi ^n\right)\nonumber \\ &\quad \quad \left. +\;\tau _n\left(\frac{1}{c}q^n(\theta ^n-\theta ^{n-1}),q^n(\theta ^n-\theta ^{n-1})\right)\right] +\tau _n(\tilde{A}\pi ^n,\pi ^n)\nonumber \\ &\quad \quad +\frac{\tau _n}{2}[(\tilde{A}\pi ^n,\pi ^n)+(\tilde{A}(\pi ^n-\pi ^{n-1}),\pi ^n-\pi ^{n-1})]\nonumber \\ &\quad =\frac{\tau _n}{2}(\tilde{A}\pi ^{n-1},\pi ^{n-1}) +\tau _n\left( \tilde{A}(\pi ^{n-1}-\pi ^n),A\nabla (\theta ^n-\theta ^{n-1})+\mathbf {b}^{n}(\theta ^n-\theta ^{n-1})\right) \nonumber \\ &\quad \quad -\tau _n\left( \frac{1}{c}(\nabla \cdot \pi ^n+q^n\theta ^{n-1}),c(\theta ^n-\theta ^{n-1})+\tau _n(\nabla \cdot \pi ^n+q^n(\theta ^n-\theta ^{n-1}))\right) \nonumber \\ &\quad \quad +\tau _n\left( \tilde{A}(\mathbf {b}^{n-1}-\mathbf {b}^n)\theta ^{n-1},\pi ^n+A\nabla (\theta ^n-\theta ^{n-1})+\mathbf {b}^n(\theta ^n-\theta ^{n-1})\right) \nonumber \\ &\quad \quad +a_n(({\sigma }^{n}_h-\hat{\sigma }^{n}_h,u^n_h-\hat{u}^n_h),(\pi ^n,\theta ^n-\theta ^{n-1})). \end{aligned}$$

Next, we estimate the terms on the right-hand side of the error equation (22).

It is clear that

$$\begin{aligned}&\tau _n(\tilde{A}(\pi ^{n-1}-\pi ^n),A\nabla (\theta ^n-\theta ^{n-1}))\\ &\quad \le \frac{\tau _n}{2}\left[\left(\tilde{A}(\pi ^n-\pi ^{n-1}),\pi ^n-\pi ^{n-1})+(A\nabla (\theta ^n-\theta ^{n-1}),\nabla (\theta ^n-\theta ^{n-1})\right)\right] \end{aligned}$$

and

$$\begin{aligned}&\tau _n\left|\left( \tilde{A}(\pi ^{n-1}-\pi ^n),\mathbf {b}^{n}(\theta ^n-\theta ^{n-1})\right) \right| +\tau _n\left| \left( \frac{1}{c}(\nabla \cdot \pi ^n+q^n\theta ^{n-1}),c(\theta ^n-\theta ^{n-1})\right. \right.\\ &\quad \quad \left.\left. +\tau _n(\nabla \cdot \pi ^n+q^n(\theta ^n-\theta ^{n-1}))\right) \right|\\ &\quad \quad +\tau _n\left|\left( \tilde{A}(\mathbf {b}^{n-1}-\mathbf {b}^n)\theta ^{n-1},\pi ^n+A\nabla (\theta ^n-\theta ^{n-1})+\mathbf {b}^n(\theta ^n-\theta ^{n-1})\right) \right|\\ &\quad \quad +|a_n(({\sigma }^{n}_h-\hat{\sigma }^{n}_h,u^n_h-\hat{u}^n_h),(\pi ^n,\theta ^n-\theta ^{n-1}))|\\ &\quad \le K\tau _n\left\{ \left(\frac{h^2}{H^2}+\frac{\tau }{H^2})^{m}\int ^{t^n}_{t^{n-1}} \left\Vert\left(\frac{\partial \sigma _h}{\partial t},\frac{\partial w_h}{\partial t}\right)\right\Vert ^2_{a_n}\text {d}t+\tau _n[\Vert \theta ^{n-1}\Vert ^2_{L^2(\varOmega )}\right.\right. \\ &\quad \quad +\Vert \nabla (\theta ^n-\theta ^{n-1})\Vert ^2_{[L^2(\varOmega )]^d}+\Vert \pi ^{n-1}\Vert ^2_{[L^2(\varOmega )]^d}+\Vert \pi ^{n}\Vert ^2_{[L^2(\varOmega )]^d}\\ &\quad \quad \left. +\;\Vert \nabla \cdot \pi ^{n-1}\Vert ^2_{L^2(\varOmega )}] \right\} +\delta [\Vert \theta ^n-\theta ^{n-1}\Vert ^2_{L^2(\varOmega )}+\tau ^2_n\Vert \nabla \cdot \pi ^{n}\Vert ^2_{L^2(\varOmega )}]. \end{aligned}$$

Substituting the above estimates into (22) and then summing it up from 1 to *n*, we get

23 $$\begin{aligned}&\Vert \pi ^{n}\Vert ^2_{[L^2(\varOmega )]^d}+\sum ^n_{j=1}\tau _j\left[ \Vert \bar{\partial }_t\theta ^j\Vert ^2_{L^2(\varOmega )} +\tau _j\Vert \nabla \bar{\partial }_t\theta ^j\Vert ^2_{[L^2(\varOmega )]^d}+\tau _j\Vert \nabla \cdot \pi ^j\Vert ^2_{L^2(\varOmega )}\right] \nonumber \\ &\quad \le K\left\{\left(\frac{h^2}{H^2}+\frac{\tau }{H^2}\right)^{m}\int ^{t^n}_{0}\left\Vert \left(\frac{\partial \sigma _h}{\partial t},\frac{\partial w_h}{\partial t}\right)\right\Vert ^2_{a_n}\text {d}t+\sum ^n_{j=1}\tau _j\left[ \Vert \pi ^{j}\Vert ^2_{[L^2(\varOmega )]^d}\right.\right. \nonumber \\ &\quad \quad \left.\left. +\Vert \theta ^{j}\Vert ^2_{L^2(\varOmega )} +\tau ^2_j\Vert \nabla \bar{\partial }_t\theta ^j\Vert ^2_{[L^2(\varOmega )]^d}\right] \right\}. \end{aligned}$$

Applying a known inequality

$$\begin{aligned} \Vert \theta ^n\Vert ^2_{L^2(\varOmega )}\le \Vert \theta ^0\Vert ^2_{L^2(\varOmega )}+\delta \sum ^n_{j=1}\tau _j\Vert \bar{\partial }_t\theta ^j\Vert ^2_{L^2(\varOmega )}+K\sum ^n_{j=1}\tau _j\Vert \theta ^j\Vert ^2_{L^2(\varOmega )} \end{aligned}$$

and discrete Gronwall’s lemma to (23), we derive that

24 $$\begin{aligned} \max _n\Vert \theta ^n\Vert _{L^2(\varOmega )}+\max _n\Vert \pi ^n\Vert _{[L^2(\varOmega )]^d}\le K\left(\frac{h^2}{H^2}+\frac{\tau }{H^2}\right)^{\frac{m}{2}}. \end{aligned}$$

Using Lemma 1, we can obtain the estimate (11). The proof of Theorem 1 is complete. $\square$

## Numerical results

As in Zhang and Yang (2011), we first consider the one dimensional convection–diffusion problem:

25 $$\begin{aligned} \frac{\partial u}{\partial t}-a\frac{\partial ^2u}{\partial x^2}-b\frac{\partial u}{\partial x}+u=f\quad x\in [0,1], \quad 0 \le t \le T. \end{aligned}$$

**Fig. 1:**
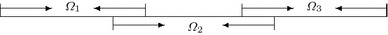
The sub-domains of $\varOmega$

We divide the domain [0, 1] into three sub-domains: $$\varOmega _1=\left[0,\frac{1}{3}+\frac{H}{2}\right],$$$$\varOmega _2=\left[\frac{1}{3}-\frac{H}{2},\frac{2 }{3}+\frac{H}{2}\right]$$ and $$\varOmega _3=\left[\frac{2 }{3}-\frac{H}{2},1\right]$$, where *H* is the overlapping degree (see Fig. 1).

We use piecewise linear polynomial spaces, set $h_u=h_\sigma =h$ and take the linear unit decomposition functions as in Zhang et al. (2011). We define the $L^2$-norm error as follows:

$$\begin{aligned} \Vert (e,E)\Vert ^2_2=\max _n\Vert u^n-u^n_h\Vert _{L^2(\varOmega )}+\max _n\Vert \sigma ^n-\sigma _h^n\Vert _{L^2(\varOmega )}, \end{aligned}$$

and the $L^\infty$-norm error

$$\begin{aligned} \Vert (e,E)\Vert _\infty =\max _n(|e^n|,|E^n|). \end{aligned}$$

**Experiment I** In this experiment, the exact solution is chosen as $u=e^t\sin ^2\pi x$. Set $T=1$, and $b=1$. For different parameters *a*, *h*, $\tau$ and the iterative number *m* at each time step, we give $L^2$-norm errors and the $L^\infty$-norm errors in Tables 1, 2 and 3. These numerical results suggest that we can get a good result for convection–diffusion problem using parallel algorithm , even iterating only one or two cycle at each time step. Moreover, these numerical results also imply that the errors caused by decomposing domain decrease as the discretization parameters *h* and $\tau$ decrease and increase as the overlapping degree *H* becomes small, which are coincided with our theoretical result.

**Table 1:** $$H=\frac{1}{6},\ h=\tau $$

*h*	*m*	$a=1$		$a=1$ *e*–2		$a=1e{-}4$	
		$\Vert \cdot \Vert _2$	$\Vert \cdot \Vert _\infty$	$\Vert \cdot \Vert _2$	$\Vert \cdot \Vert _\infty$	$\Vert \cdot \Vert _2$	$\Vert \cdot \Vert _\infty$
$\frac{1}{48}$	$*$	$2.9866$ *e*–2	$1.9250$ *e*–1	$8.6118$ *e*−3	$2.6493$ *e*–2	$7.4344$ *e*−3	$1.5421$ *e*–2
$\frac{1}{48}$	1	$4.0090$ *e*–2	$2.0819$ *e*–1	$8.5534e{-}3$	$2.5531$ *e*–2	$7.4495e{-}3$	$1.4833$ *e*–2
$$\frac{1}{48}$$	2	$$4.0086 $$ *e*–2	$$1.7485$$ *e*–1	$$8.6384e{-}3$$	$$2.5532$$ *e*–2	$$7.4785e{-}3$$	$$1.5375$$ *e*–2
$\frac{1}{48}$	3	$4.1043$ *e*–2	$1.7967$ *e*–1	$8.6403e{-}3$	$2.5532$ *e*–2	$7.4790e{-}3$	$1.5379$ *e*–2
$$\frac{1}{48}$$	4	$$4.1164$$ *e*–2	$$1.7947$$ *e*–1	$$8.6403e{-}3$$	$$2.5532 $$ *e*–2	$$7.4790e{-}3$$	$$1.5379 $$ *e*–2
$\frac{1}{96}$	$*$	$1.4800$ *e*–2	$9.5488$ *e*–2	$4.9541e{-}3$	$2.5220$ *e*–2	$3.6348e{-}3$	$7.4154e{-}3$
$\frac{1}{96}$	1	$1.7513$ *e*–2	$1.0523$ *e*–1	$4.9351e{-}3$	$2.4967$ *e*–2	$3.6397e{-}3$	$7.3584e{-}3$
$\frac{1}{96}$	2	$1.7951$ *e*–2	$9.1683$ *e*–2	$4.9457e{-}3$	$2.4967$ *e*–2	$3.6420e{-}3$	$7.4145e{-}3$
$\frac{1}{96}$	3	$1.8186$ *e*–2	$9.3327$ *e*–2	$4.9458e{-}3$	$2.4967$ *e*–2	$3.6420e{-}3$	$7.4147 e{-}3$
$$\frac{1}{96}$$	4	$$1.8219$$ *e*–2	$$9.3540$$ *e*–2	$$4.9458e{-}3$$	$$2.4967 $$ *e*–2	$$3.6420e{-}3$$	$$7.4147e{-}3$$
$$\frac{1}{192}$$	$$*$$	$$ 7.5407e{-}3$$	$$ 4.7396$$ *e*–2	$$2.9959e{-}3$$	$$2.0611$$ *e*–2	$$1.8231e{-}3$$	$$4.2963e{-}3$$
$\frac{1}{192}$	1	$7.9423e{-}3$	$5.1561$ *e*–2	$2.9899e{-}3$	$2.0539$ *e*–2	$1.8239e{-}3$	$4.2920e{-}3$
$\frac{1}{192}$	2	$8.1633e{-}3$	$4.7424$ *e*–2	$2.9912e{-}3$	$2.0539$ *e*–2	$1.8240e{-}3$	$4.2920e{-}3$
$\frac{1}{192}$	3	$8.1986e{-}3$	$4.7096$ *e*–2	$2.9912e{-}3$	$2.0539$ *e*–2	$1.8240e{-}3$	$4.2920e{-}3$
$$\frac{1}{192}$$	4	$$8.2016 e{-}3$$	$$4.7458$$ *e*–2	$$2.9912e{-}3$$	$$ 2.0539$$ *e*–2	$$1.8240e{-}3$$	$$4.2920e{-}3$$

* The numerical results by least-squares algorithm

**Table 2:** $$H=\frac{1}{12},\ h=\tau $$

*h*	*m*	$a=1$		$a=1$ *e*–2		$a=1e{-}4$	
		$\Vert \cdot \Vert _2$	$\Vert \cdot \Vert _\infty$	$\Vert \cdot \Vert _2$	$\Vert \cdot \Vert _\infty$	$\Vert \cdot \Vert _2$	$\Vert \cdot \Vert _\infty$
$$\frac{1}{48}$$	$$*$$	$$2.9866$$ *e*–2	$$1.9250$$ *e*–1	$$8.6118e{-}3$$	$$2.6493$$ *e*–2	$$7.4344e{-}3$$	$$ 1.5421$$ *e*–2
$$\frac{1}{48}$$	1	$$ 8.4704$$ *e*–2	$$2.2515$$ *e*–1	$$8.7074e{-}3$$	$$2.4202$$ *e*–2	$$7.5866e{-}3$$	$$1.6164$$ *e*–2
$\frac{1}{48}$	2	$8.0447$ *e*–2	$1.9139$ *e*–1	$8.7352e{-}3$	$2.4202$ *e*–2	$7.5212e{-}3$	$1.5371$ *e*–2
$\frac{1}{48}$	3	$7.9085$ *e*–2	$1.8885$ *e*–1	$8.7459e{-}3$	$2.4202$ *e*–2	$7.5203e{-}3$	$1.5388$ *e*–2
$\frac{1}{48}$	4	$7.7713$ *e*–2	$1.8467$ *e*–1	$8.7464e{-}3$	$2.4202$ *e*–2	$7.5202e{-}3$	$1.5389$ *e*–2
$$\frac{1}{96}$$	$$*$$	$$1.4800$$ *e*–2	$$9.5488$$ *e*–2	$$4.9541e{-}3$$	$$2.5220$$ *e*–2	$$3.6348e{-}3$$	$$ 7.4154e{-}3$$
$\frac{1}{96}$	1	$3.7063$ *e*–2	$1.0595$ *e*–1	$4.9336e{-}3$	$2.4609$ *e*–2	$3.6506e{-}3$	$7.4109e{-}3$
$\frac{1}{96}$	2	$3.5576$ *e*–2	$8.4116$ *e*–2	$4.9480e{-}3$	$2.4609$ *e*–2	$3.6484e{-}3$	$7.4152e{-}3$
$$\frac{1}{96}$$	3	$$3.5206$$ *e*–2	$$8.2344$$ *e*–2	$$4.9480e{-}3$$	$$2.4609 $$ *e*–2	$$3.6481e{-}3$$	$$7.4153e{-}3$$
$$\frac{1}{96}$$	4	$$3.4930$$ *e*–2	$$8.0713$$ *e*–2	$$4.9480e{-}3$$	$$2.4609 $$ *e*–2	$$3.6481e{-}3$$	$$7.4153e{-}3$$
$\frac{1}{192}$	$*$	$7.5407e{-}3$	$4.7396$ *e*–2	$2.9959e{-}3$	$2.0611$ *e*–2	$1.8231e{-}3$	$4.2963e{-}3$
$\frac{1}{192}$	1	$1.3608$ *e*–2	$5.2234$ *e*–2	$2.9850e{-}3$	$2.0431$ *e*–2	$1.8251e{-}3$	$4.2847e{-}3$
$\frac{1}{192}$	2	$1.3257$ *e*–2	$4.1956$ *e*–2	$2.9875e{-}3$	$2.0431$ *e*–2	$1.8249e{-}3$	$4.2847e{-}3$
$\frac{1}{192}$	3	$1.3210$ *e*–2	$4.2262$ *e*–2	$2.9875e{-}3$	$2.0431$ *e*–2	$1.8249e{-}3$	$4.2847e{-}3$
$$\frac{1}{192}$$	4	$$1.3183 $$ *e*–2	$$4.2067$$ *e*–2	$$2.9875e{-}3$$	$$2.0431$$ *e*–2	$$1.8249e{-}3$$	$$4.2847e{-}3$$

* The numerical results by least-squares algorithm

**Table 3:** $$H=\frac{1}{24},\ h=\tau $$

*h*	*m*	$a=1$		$a=1$ *e*–2		$a=1e{-}4$	
		$\Vert \cdot \Vert _2$	$\Vert \cdot \Vert _\infty$	$\Vert \cdot \Vert _2$	$\Vert \cdot \Vert _\infty$	$\Vert \cdot \Vert _2$	$\Vert \cdot \Vert _\infty$
$\frac{1}{48}$	$*$	$2.9866$ *e*–2	$1.9250$ *e*–1	$8.6118e{-}3$	$2.6493$ *e*–2	$7.4344e{-}3$	$1.5421$ *e*–2
$$\frac{1}{48}$$	1	$$ 2.0573$$ *e*–1	$$4.6896$$ *e*–1	$$9.1964e{-}3$$	$$ 2.8774$$ *e*–2	$$8.0709e{-}3$$	$$2.1458$$ *e*–2
$$\frac{1}{48}$$	2	$$2.0179 $$ *e*–1	$$4.5896$$ *e*–1	$$8.9982e{-}3 $$	$$2.1092 $$ *e*–2	$$7.6579e{-}3$$	$$1.5340$$ *e*–2
$$\frac{1}{48}$$	3	$$2.0061 $$ *e*–1	$$4.5878$$ *e*–1	$$9.0836e{-}3 $$	$$2.2172 $$ *e*–2	$$7.6797e{-}3$$	$$1.5379$$ *e*–2
$$\frac{1}{48}$$	4	$$1.9834 $$ *e*–1	$$4.5297$$ *e*–1	$$9.1164e{-}3 $$	$$2.2495 $$ *e*–2	$$7.6884e{-}3$$	$$1.5387$$ *e*–2
$$\frac{1}{96}$$	$$*$$	$$1.4800e{-}2$$	$$9.5488e{-}2$$	$$ 4.9541e{-}3$$	$$2.5220e{-}2$$	$$3.6348e{-}3$$	$$7.4154e{-}3$$
$\frac{1}{96}$	1	$1.1518$ *e*–1	$2.8223$ *e*–1	$4.8606e{-}3$	$2.3682e{-}2$	$3.6679e{-}3$	$7.6455e{-}3$
$\frac{1}{96}$	2	$1.1342$ *e*–1	$2.7485$ *e*–1	$4.8758e{-}3$	$2.3682e{-}2$	$3.6564e{-}3$	$7.4153e{-}3$
$\frac{1}{96}$	3	$1.1266$ *e*–1	$2.7333$	$4.8861e{-}3$	$2.3682e{-}2$	$3.6596e{-}3$	$7.4153e{-}3$
$\frac{1}{96}$	4	$1.1186$ *e*–1	$2.6995$ *e*–1	$4.8874e{-}3$	$2.3682e{-}2$	$3.6598e{-}3$	$7.4153e{-}3$
$\frac{1}{192}$	$*$	$7.5407e{-}3$	$4.7396e{-}2$	$2.9959e{-}3$	$2.0611e{-}2$	$1.8231e{-}3$	$4.2963e{-}3$
$$\frac{1}{192}$$	1	$$5.5060e{-}2$$	$$1.4802$$ *e*–1	$$2.9586e{-}3 $$	$$2.0194e{-}2$$	$$1.8251e{-}3$$	$$4.2712e{-}3$$
$$\frac{1}{192}$$	2	$$5.4280e{-}2$$	$$1.4441$$ *e*–1	$$2.9635e{-}3$$	$$2.0194e{-}2$$	$$1.8259e{-}3$$	$$4.2712e{-}3 $$
$$\frac{1}{192}$$	3	$$5.3930e{-}2$$	$$1.4334$$ *e*–1	$$2.9641e{-}3$$	$$2.0194e{-}2$$	$$1.8261e{-}3$$	$$4.2712e{-}3 $$
$\frac{1}{192}$	4	$5.3683e{-}2$	$1.4217$ *e*–1	$2.9641e{-}3$	$2.0194e{-}2$	$1.8261e{-}3$	$4.2712e{-}3$

* The numerical results by least-squares algorithm

**Experiment II** As in Zhang and Yang (2011), we select the right-hand side function with complex structure and the initial condition as follows:

$$ \left\{ \begin{array}{l}f(x,t)=100e^{t-\frac{x}{5}}\cos (8\pi xt)\sin ^2(7\pi x),\\ u^0(x)=0. \end{array} \right.$$

**Fig. 2:**
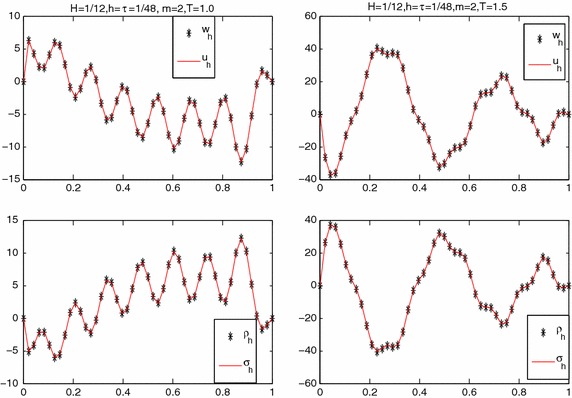
Numerical results at time $T=1.0,\ 1.5$

**Fig. 3:**
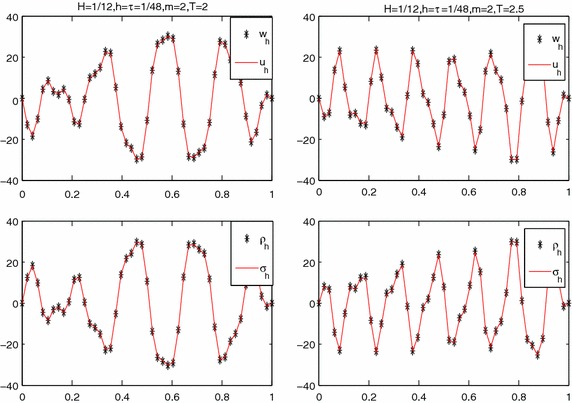
Numerical results at time $T=2.0,\ 2.5$

Choosing $H=1/12,\ h=\tau =1/48$, $b=1,$ and $a=1e{-}4,$ we observe numerical results at different time (see Figs. 2, 3). We use “ * ” to denote $u_h$ and $\sigma _h$, the values of the parallel algorithm and use “ - ” to denote $w_h$ and $\varrho _h$, the values of least-squares algorithm. These figures clearly show that $u_h,\ \sigma _h$ approximate to $w_h$ and $\varrho _h$ at different time, respectively, which is coincided with our theoretical analysis.

**Fig. 4:**
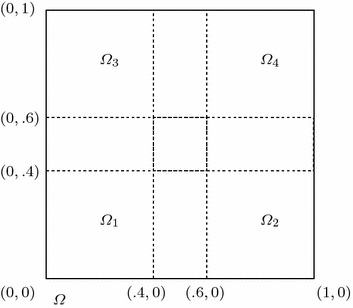
The sub-domains of $\varOmega$

Next, we consider the two dimensional convection–diffusion problem:

26 $$\begin{aligned} \left\{ \begin{aligned}&\frac{\partial u}{\partial t}+\nabla \cdot \varvec{\sigma }+u=f, \quad x\in \varOmega ,\,0<t<T,\\ &{\varvec{\sigma }}+\mathbf {A}\nabla u+\mathbf {b}u=0,\quad x\in \varOmega ,\,0<t<T, \end{aligned}\right. \end{aligned}$$

where $\varOmega =[0,1]\times [0,1],\,\mathbf {A}=a\mathbf E$, $\mathbf E$ is the unit matrix, and $\mathbf b =(1,1)^T$. We divide $\varOmega$ into four sub-domains: $\varOmega _1=[0,0.6]\times [0,0.6]$, $\varOmega _2=[0.4,1]\times [0,0.6]$, $\varOmega _3=[0,0.6]\times [0.4,1]$, $\varOmega _4=[0.4,1]\times [0.4,1]$, see Fig. 4.

In this section, we use piecewise linear polynomial spaces. And We take the linear unit decomposition functions $\{\varphi _i\}^4_{i=1}$ as follows:

$$\begin{aligned} \varphi _1(x,y)&=\left\{ \begin{array}{ll} \displaystyle 1, &{}(x,y)\in [0,0.4]\times [0,0.4],\\ \displaystyle 3-5y,&{}(x,y)\in [0,0.4]\times [0.4,0.6],\\ \displaystyle 3-5x,&{}(x,y)\in [0.4,0.6]\times [0,0.4],\\ \displaystyle \frac{3}{2}-\frac{5}{4}(x+y),&{}\text {otherwise}, \end{array}\right. \\ \varphi _2(x,y)&=\left\{ \begin{array}{ll} \displaystyle 1, &{}(x,y)\in [0.6,1]\times [0,0.4],\\ \displaystyle 3-5y,&{}(x,y)\in [0.6,1]\times [0.4,0.6],\\ \displaystyle 5x-2,&{}(x,y)\in [0.4,0.6]\times [0,0.4],\\ \displaystyle \frac{1}{4}-\frac{5}{4}(x-y),&{}\text {otherwise}, \end{array}\right. \\ \varphi _3(x,y)&=\left\{ \begin{array}{ll} \displaystyle 1, &{}(x,y)\in [0,0.4]\times [0,0.4],\\ \displaystyle 5y-2,&{}(x,y)\in [0,0.4]\times [0.4,0.6],\\ \displaystyle 3-5x,&{}(x,y)\in [0.4,0.6]\times [0.6,1],\\ \displaystyle \frac{1}{4}+\frac{5}{4}(y-x),&{}\text {otherwise}, \end{array}\right. \\ \varphi _4(x,y)&=\left\{ \begin{array}{ll} \displaystyle 1, &{}(x,y)\in [0.6,1]\times [0.6,1],\\ \displaystyle 5y-2,&{}(x,y)\in [0.6,1]\times [0.4,0.6],\\ \displaystyle 5x-2,&{}(x,y)\in [0.4,0.6]\times [0.6,1],\\ \displaystyle \frac{5}{4}(x+y)-1,&{}\text {otherwise}. \end{array}\right. \end{aligned}$$

**Experiment III ** Here we still select the same right-hand side function with complex structure and the initial condition as in Zhang and Yang (2011),

$$\left\{ \begin{array}{l}f(x,t)=e^{y^3-x^2-2t}\sin (3\pi x-6y+t^2)\cos (4\pi yt),\\ u^0(x)=0. \end{array} \right.$$

**Fig. 5:**
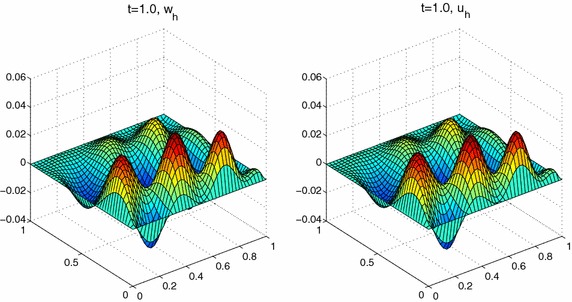
The values of $w_h$ and $u_h$ at $T=1.0$

**Fig. 6:**
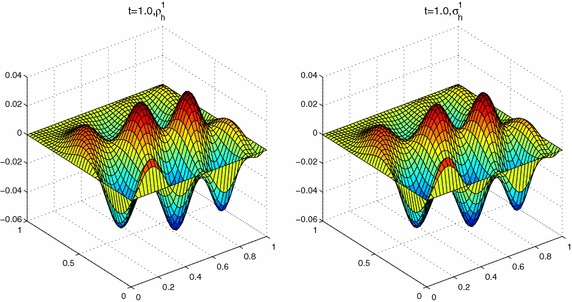
The values of $\rho ^1_h$ and $\sigma ^1_h$ at $T=1.0$

**Fig. 7:**
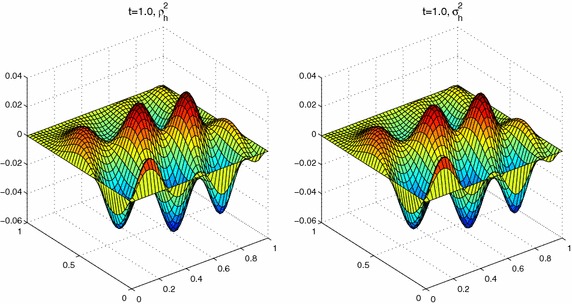
The values of $\rho ^2_h$ and $\sigma ^2_h$ at $T=1.0$

Set $$H=0.2$$, $$h=\tau =1/40$$, and $$a=1e{-}2$$, $$ T=1.0$$, $$ m=1$$. We can get Figs. 5, 6 and 7. These results suggest that the values $$u_h$$, $$ \varvec{\sigma }_h=(\sigma ^1_h,\sigma ^2_h)$$ by parallel algorithm approximate to $$w_h$$ and the values $$\rho _h=(\rho ^1_h,\rho ^2_h)$$ by least-squares scheme respectively, which implies that our method is valid for two-dimensional problem.

## Conclusions

In this paper, combined subspace correction method with least-squares mixed element procedure, a new class of parallel domain decomposition algorithm is proposed to solve convection–diffusion problem. The convergence of approximate solution, and the dependence of the convergent rate on the spacial mesh size, time increment, iteration number and sub-domains overlapping degree are studied. Both theoretical analysis and numerical experiments indicate the full parallelization of the algorithms and very good approximate property.

In fact, though we consider the convection–diffusion problem in this paper, we can extend our method to other complex problems, e.g. saltwater intrusion problem, aerodynamic problems, nuclear waste disposal, etc., which are our future work.
